# A novel gene, *CaATHB-12*, negatively regulates fruit carotenoid content under cold stress in *Capsicum annuum*

**DOI:** 10.29219/fnr.v64.3729

**Published:** 2020-12-28

**Authors:** Rui-Xing Zhang, Wen-Chao Zhu, Guo-Xin Cheng, Ya-Nan Yu, Quan-Hui Li, Saeed ul Haq, Fazal Said, Zhen-Hui Gong

**Affiliations:** 1 College of Horticulture, Northwest A&F University, Yangling, Shaanxi, P.R. China; 2 Guizhou Institute of Pepper, Guiyang, P.R. China; 3 Department of Agriculture, Abdul Wali Khan University, Mardan, Paksitan

**Keywords:** pepper, carotenoids, CaATHB-12 gene, cold stress, transgenic Arabidopsis

## Abstract

**Background:**

Carotenoids, the secondary metabolites terpenoids, are the largest factors that form the fruit color. Similar to flavonoids, they are not only safe and natural colorants of fruits but also play a role as stress response biomolecules.

**Methods:**

To study the contribution of the key genes in carotenoids biosynthesis, fruit-color formation, and in response to cold stress, we characterized the key regulatory factor *CaATHB-12* from the HD-ZIP I sub-gene family members in pepper.

**Results:**

Cold stress enhanced carotenoid accumulation as compared with the normal condition. *CaATHB-12* silencing through virus-induced gene silencing changed the fruit color by regulating the carotenoid contents. *CaATHB-12* silencing increased the antioxidant enzyme activities in the fruits of pepper, exposed to cold stress, whereas *CaATHB-12* overexpression decreased the activities of antioxidant enzymes in the transgenic *Arabidopsis* lines, exposed to cold stress, suggesting that *CaATHB-12* is involved in the regulation of cold stress in the pepper fruits.

**Conclusion:**

Our research will provide insights into the formation of fruit color in pepper and contribution of *CaATHB-12* in response to cold stress. Further study should be focused on the interaction between *CaATHB-12* and its target gene.

## Popular scientific summary

- Carotenoids contents play a role not only in pepper fruit color development but also in stress response.
- The novel gene, *CaATHB-12*, regulated the fruit carotenoid contents.
- Silencing of *CaATHB-12* in pepper increased the antioxidant enzymes under cold stress.
- Overexpression of *CaATHB-12* in *Arabidopsis* decreased the level of antioxidant enzymes activities.

The growth and development of pepper are often affected by adversities, of which chilling is an important factor (1). Due to significant lipid degradation, peppers are more susceptible to low temperatures than tomatoes, potatoes, cucumbers, and corns (2). Generally, the growth of pepper is affected when the temperature is below 12°C (3, 4). Cold stress results in the severe membrane lipid peroxidation due to the production of reactive oxygen species (ROS) and generation of malondialdehyde (MDA) in the cell. The ROS destroys the morphological structures and physiological metabolism in the pepper. To mitigate the injury, the plant removes excess ROS by improving activities of antioxidant enzymes such as superoxide dismutase (SOD), catalase (CAT), and peroxidase (POD) (5).

Many homeobox genes encode transcription factors that act as major regulators in growth and development of both the plants and animal, including humans (6–8). They are essential from the early stages of embryonic development to the latest stages of cell differentiation (9). The pressure-sensitive HD-ZIP protein, belonging to subfamily I, has been reported wildly in recent years (9). It contains a plant-specific TFs (transcription factors) with the highly conserved and unique sequence and plays a key role in the growth and development of plants (10). *ATHB-12* gene, a member of the HD-ZIP I gene family, is involved in response to a variety of stresses during the growth of plants. *ATHB-12* regulates plant growth and development in various environmental stresses, including drought (11) and cold (9). In maize, modified expression of *Zmhdz10* as an *HD-Zip I* gene regulates response to low temperature and abscisic acid (ABA) (12). Similar findings were reported in the model plant *Arabidopsis*, where *ATHB-7* overexpression promoted leaf development and increased chlorophyll content and photosynthesis. *ATHB-7* also reduced the stomatal conductance in mature plants and delayed plant senescence in response to ABA, low temperature, and other environmental stresses (13).

During the ripening of fruits, one of the characteristic change is the fruit color, which is closely related to carotenoid contents (14, 15). Carotenoids are also thought to be associated with reduced risk of several chronic health disorders, including some forms of cancer, heart diseases, and eyes degeneration (16, 17). Carotenoids, also called tetraterpenoids, accumulate in the plastids of the cell, which not only prevent photooxidative damage in the plant (18, 19) but also *benefit* to humans who often eat the carotenoids-rich food for enhanced immunity (20–22). In *Dunaliella*, low temperature induced the accumulation of carotenoids and carotenoid-binding proteins (23). Similarly, in California poppy, the total carotenoids content was reduced by the silencing of *PDS*, *ZDS*, and *ZEP* genes, thereby resulted in a color change (10). Therefore, carotenoid accumulation is important for the improvement of fruit quality and resistance to adversities.

Earlier the HD-ZIP TFs are widely involved in flower and fruit development and organ maturation and senescence (12, 24, 25). For example, *LeHB-1*, a tomato homeobox protein, is involved in the control of tomato fruit ripening through reduced *LeACO1* mRNA levels (26). The sunflower HD-Zip transcription factor *HAHB4* encodes components of photosystem I (*LHCa*) and photosystem II (*PSBx*) genes related to the chlorophyll biosynthesis. Many of the chlorophyll-binding proteins are apparently downregulated by *HAHB4*, and the content of chlorophylls a and b and carotenoids was decreased in transgenic *Arabidopsis* plants (27). Other HD-Zip TFs have been suggested to influence the accumulation of anthocyanin (28–30). For example, *ANTHOCYANINLESS2* (*AtANL2*) was involved in the tissue-specific accumulation of anthocyanin. Histological observations of the *anl2* mutant revealed that the anthocyanin accumulation was greatly suppressed in subepidermal cells (31). Although several HD-Zip proteins have been well characterized in different plants, the functions of HD-Zip family members are still unknown in pepper. Previously, *ATHB-12* gene has been studied in response to drought stress in the model plant *Arabidopsis* (32), and many of the studies have focused on salt stress and ABA induction (33). To date, a little is known about the role of the *ATHB-12* gene in fruit color development and response to cold stress in the fruits of pepper. Hence, we employed virus-induced gene silencing (VIGS) to silence the *ATHB-12* gene in pepper, which was selected from the pepper transcriptome database to lower the expression of *ATHB-12*. We investigated the effect of *ATHB-12* expression on fruit-color formation, carotenoids biosynthesis, and response to low temperature in pepper. Similarly, the function of this gene was also validated in the transgenic *Arabidopsis* in response to low temperature. Stress- and antioxidant-related genes, secondary metabolites, such as flavonoids and phenolic compounds, and antioxidant enzyme activities were studied. The results of this study will provide insights into the mechanism of pepper carotenoid biosynthesis and will provide a basis for the breeding of fruit color and resistance to cold stress in pepper and other important crops.

## Materials and methods

### Plants and cultivation

Pepper cultivar AA3 (a tolerant storage cultivar) was provided by the Capsicum Research Group, College of Horticulture, Northwest A&F University, P.R. China. Seeds were germinated according to the method of Wang et al. (34) with little modifications. The seeds were treated with warm water (55°C) for 20 min, soaked in water for 5 h at 28°C, and then covered with a wet cotton cloth and placed in the dark in a growth chamber. When the seeds were approximately 80% germinated after 4 days, they were transferred to pots and raised as seedlings. When the seedlings reached 8–10 true leaves, they were taken and transplanted into plastic high-tunnels. The fruits (all same age) on the 35th day after anthesis (green mature stage) were picked and transferred to the laboratory for further experiments according to a method described by Tian et al. (35).

### Subcellular localization

The open reading frame (ORF) fragment (732 bp) of *CaATHB-12* without a stop codon was cloned from pepper cDNA using the specific primers pair (Supplementary Table 1), with the restriction enzymes sites *Xba*I and *Kpn*I. Then, the PCR-amplified *CaATHB-12* fragment was cloned into a pVBG2307:GFP (green fluorescent protein) vector and a pVBG2307:GFP vector without the *CaATHB-12* gene used as a control. All recombinant fusion vectors were transient over-expressed in the *Nicotiana benthamiana* leaves using the transformation of *Agrobacterium tumefaciens* strain GV3101 (36).

### Construction of tobacco rattle virus plasmids and generation of CaATHB-12-silenced fruits

The optimal tobacco rattle virus (TRV)-based VIGS system was employed to silence the *ATHB-12* expression in the pepper line AA3. TRV has bipartite RNA; RNA1 (TRV1) and RNA2 (TRV2) sequences were used independently as vectors in the plant cells. The TRV2 vector carried heterologous nucleic acid for delivery into a plant. According to the structure of the TRV, special primers of *CaATHB-12* and Ca*PDS* were designed in the nonconservative domain of ORFs, which transferred the target genes into the TRV vector to generate TRV2:*CaATHB-12* and TRV2:Ca*PDS* (the positive control) (Supplementary Fig. 1). The empty vector (TRV:00) was used as a negative control. The TRV1, TRV2, and TRV2:*CaATHB-12* vectors were individually transformed into the *A. tumefaciens* strain GV3101. The *Agrobacterium* strain GV3101 carrying TRV1 was separately mixed with TRV2 and the empty vectors TRV2:00 and TRV2:*CaATHB-12* at a 1:1 ratio. The suspensions of the *Agrobacterium* inoculation containing TRV1, TRV2, and TRV2:*CaATHB-12* (OD600 = 1.0) were infiltrated into the pepper fruits using a 1.0-mL sterilized syringe without a needle. The fruits were placed on sterilized filter papers on a plate and covered with food grade plastic film. The plates were placed in a dark chamber (18°C and 35% relative humidity) for 2 days, and then the treated fruits were transferred into a growth chamber at 23°C/20°C with a 16 h light/8 h dark photoperiod cycle at 35% relative humidity. The control fruits (TRV:00) and silenced fruits (TRV2:*CaATHB-12*) were used for gene expression analysis 15 days after inoculation.

### Generation of CaATHB-12 transgenic Arabidopsis lines

The full length of the *CaATHB-12* ORF was cloned from pepper cDNA using the specific primer pair (Supplementary Table 1) with the restriction enzymes sites *Xba*I and *Kpn*I. The PCR-amplified products were cloned into the plant expression vector pVBG2307. The recombinant fusion vector was transformed into *Arabidopsis thaliana using* the *A. tumefaciens* strain GV3101 for transforma-tion (37). Transgenic plants were grown on Murashige and Skoog (MS) medium containing 50 mM/L kanamy-cin and PCR verification. T3 seeds were used for further experiments.

### Stress treatment

Cold stress was applied to the pepper fruits according to the methods of Cabello et al. (38) with little modifications. When photo-bleaching was observed on the fruit of TRV2:*CaPDS*, the silenced pepper seedling with TRV2:*CaATHB-12* was exposed to cold stress (4°C) for 1 day. Fruits were sampled at 0, 6, 12, and 24 h. The design used was completely randomized within the growth chamber with three biological replicates.

### RNA extraction and quantitative real-time PCR

Total RNA was extracted using a plant RNA Kit (Omega Bio Tek, USA) according to the manufacturer’s instructions, and then reverse transcription was performed using the Prime-script™ first-strand cDNA synthesis kit (TaKaRa, Dalian, China). A list of the CDS sequences of *CaATHB-12* and the primers pair used for quantitative real-time PCR (qRT-PCR) are given in Supplementary Tables 1 and 2. qRT-PCR was performed according to the method of Ali et al. (39). The ubiquitin-conjugating gene *CaUbi3* (AY486137) was used as the reference gene for the pepper (40), while actin gene *AtActin2* (At3g18780) was used as the reference gene for *Arabidopsis*. The relative expression levels were calculated by the 2^−∆∆CT^ method (41). All the samples were obtained in triplicate, and each treatment had at least three independent biological replicates.

### Measurement of pigment content

A 0.2 g of fruits was collected for the measurement of chlorophyll levels. First, fruits were grinded into pieces and soaked in 10 mL acetone for 48 h. Extracts were centrifuged at 5,000 rpm for 15 min at 4°C. According to the method of Porra et al. (42), absorbance of the supernatants was determined at 644, 663, and 440 nm for the measurement of chlorophyll and carotenoid contents using a spectrophotometer.

### Measurements of antioxidant enzymes, total phenols, flavonoids, and malondialdehyde contents

The determination of total phenols and flavonoids was slightly modified with reference to the method of Wilson (43). We used (OD280/g) and (OD325/g) to calculate the relative amounts of total phenols and flavonoids, respectively.

To measure the SOD activity, 0.5 g of fresh samples was grinded in a mortar and pestle with 5 cm^3^ of phosphate buffer solution (PBS) containing 50 mM PBS, 25 mM nitro tetrazolium blue chloride, 0.003 mM riboflavin, and 0.1 mM ethylene diamine tetra acetic acid at pH 7.8. The homogenates were centrifuged at 13,000 rpm at 4°C for 15 min. The supernatants were exposed to an irradiance of 500 μmol m^-2^ s^-1^ for 20 min. Activity of SOD was quantified spectrophotometrically at 560 nm (*A*_560_ of the control containing water instead of the supernatant was determined in darkness). The SOD activity was calculated based on Dionisio-Sese and Tobita (44).

To measure the POD activity, 0.1 g of fresh samples was grinded in a mortar and pestle with 5 cm^3^ of PBS (20 mM, pH 6.0). Homogenates were centrifuged at 13,000 rpm at 4°C for 10 min. The supernatants were exposed to an irradiance of 500 μmol m^−2^ s^−1^ for 20 min. Activity of POD was quantified spectrophotometrically at 470 nm, and absorbances were recorded every 30 s. The POD activity was calculated by the method of Dionisio-Sese and Tobita (44).

For measuring the MDA content, 0.2 g of the samples was ground in liquid nitrogen through pestle and mortar and subsequently 5 cm^3^ of ice-cold 10% (m/v) trichloroacetic acid was added. Content of MDA was measured following the protocol of Dionisio-Sese and Tobita (44) with minor modifications. Briefly, the homogenates were centrifuged at 13,000 rpm for 15 min, and the supernatants were added to the same volume of a 10% (m/v) thiobarbituric acid solution containing 0.6% (m/v) trichloroacetic acid. The mixtures were heated at 100°C for 20 min, and the reaction was rapidly halted by placing the mixtures into an ice bath. The cooled reaction solutions were then centrifuged at 13,000 rpm for 10 min, and the absorbance of the supernatants was measured at 450, 532, and 600 nm.

CAT activity was measured by the method of Beers and Sizer (45). Lyophilized fruit (1.00 g) powder was ground in a mortar and homogenized with 5 mL ice-cold extraction buffer (100 mM potassium phosphate buffer, pH 7.5, 1 mM EDTA (Ethylene Diamine Tetraacetic Acid), and 4% polyvinylpyrrolidone). The homogenate was centrifuged at 13,000 rpm for 20 min at 4°C. The supernatant fraction was used as a crude extract for enzyme activity assays. The reaction system is as follows: 0.1 mL crude extraction enzyme solution + 0.7 mL 50 mM PBS (pH 7.0) + 0.2 mL 200 mM H_2_O_2_.

Glutathione peroxidase (GPX) activity was measured following the methods of Flohé and Günzler (46). The reaction system is as follows: 0.4 mL crude extraction enzyme solution + 0.4 mL 1 mM GSH + 0.2 mL 1.5 mM H_2_O_2_ (37°C) + 4 mL 0.61 mM trichloroacetic acid + 2.5 mL 0.32 M Na_2_HPO_4_ + 0.5 mL DTNB (0.04% DTNB (5, 5’-dithiobis-(2-nitrobenzoic acid)), 1% trisodium citrate).

## Results

### Subcellular localization of CaATHB-12 proteins

**Fig. 1:**
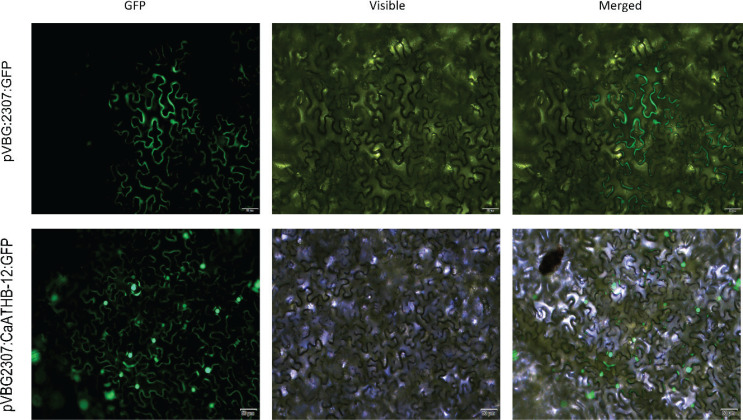
Subcellular localization of the pVBG2307:*CaATHB-12*:GFP fusion protein in *N. benthamiana* leaves, pVBG2307:GFP, was used as control. The fluorescence was observed under bright and fluorescence field. GFP: green fluorescence of green fluorescence protein (GFP). The scale bar represents 50 μm.

The subcellular location of a protein is an important characteristic with functional implications in cell (47). To determine whether the *CaATHB-12* protein has a function in life progress, subcellular localization of *CaATHB-12* was predicted using WoLF PSORT (https://wolfpsort.hgc.jp/), which predicted that *CaATHB-12* was localized mainly in the nucleus (Supplementary Table 3). To confirm this, subcellular localization was characterized by transient expression of the *CaATHB-12* gene and GFP in *N. benthamiana* leaves using the *Agrobacterium*-mediated transformation technique following the methods of Jin et al. (48). We expressed GFP-tagged *CaATHB-12* under a strong promoter 35S (pVBG2307:*CaATHB-12*) and found that the tagged protein was uniformly distributed in the nucleus. However, the control pVBG2307:GFP localized throughout the cell (Fig. 1).

### Effect of CaATHB-12 silencing on the pepper fruit color

**Fig. 2:**
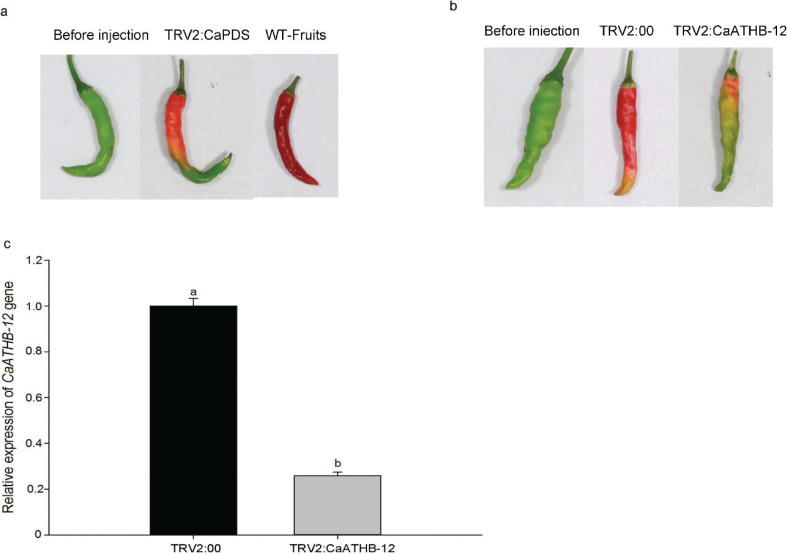
TRV-mediated silencing of *CaATHB-12* in pepper fruits. (a) Phenotypes of pepper fruits infiltrated with TRV2:*CaPDS* construct (the positive control). Before injection: the fruit on the 35th day after anthesis, when it is still in the green mature stage; WT-fruit: the phenotype of fruits that were not injected with the TRV vector carrying the *CaATHB-12* gene after fruits were kept in growth chambers for 15 days. (b) *CaATHB-12*-silencing fruits exhibiting varying phenotypes in comparison to the negative control with TRV2:00 vector. (c) Silencing efficiency in the seedlings with TRV2:*CaATHB-12* vector. The silencing efficiency was analyzed by qRT-PCR. The experiment was conducted after oranging was presented on the fruits of positive control with TRV2:*CaPDS*. Values are means ± SD from three separate experiments, and the letters show the significance level at *α* = 0.05.

To understand the role of *CaATHB-12* in carotenoid biosynthesis, VIGS technology was used in the green fruits of pepper line AA3. Compared with the control fruits, different colors were observed in the fruits that were treated with the TRV2:*CaATHB-12* gene, after 15 days of inoculation. Among them, fruit of TRV2:*CaPDS* gene showed a slightly orange color after 15 days of injection, while the control fruits showed the change from green to red color (Fig. 2a and Supplementary Fig. 2). The yellow-orange color was observed in the pepper of the TRV2:00, while pepper fruits of TRV2:*CaATHB-12* showed a little pale yellow at the fruits’ stalks (Fig. 2b and Supplementary Fig. 2). These results of the expression suggested the reliability of the VIGS, which was verified by the 75% silencing efficiency of the *CaATHB-12* gene expression after 15 days of injection (Fig. 2c).

### Effect of low temperature on CaATHB-12-silenced fruit color

**Fig. 3:**
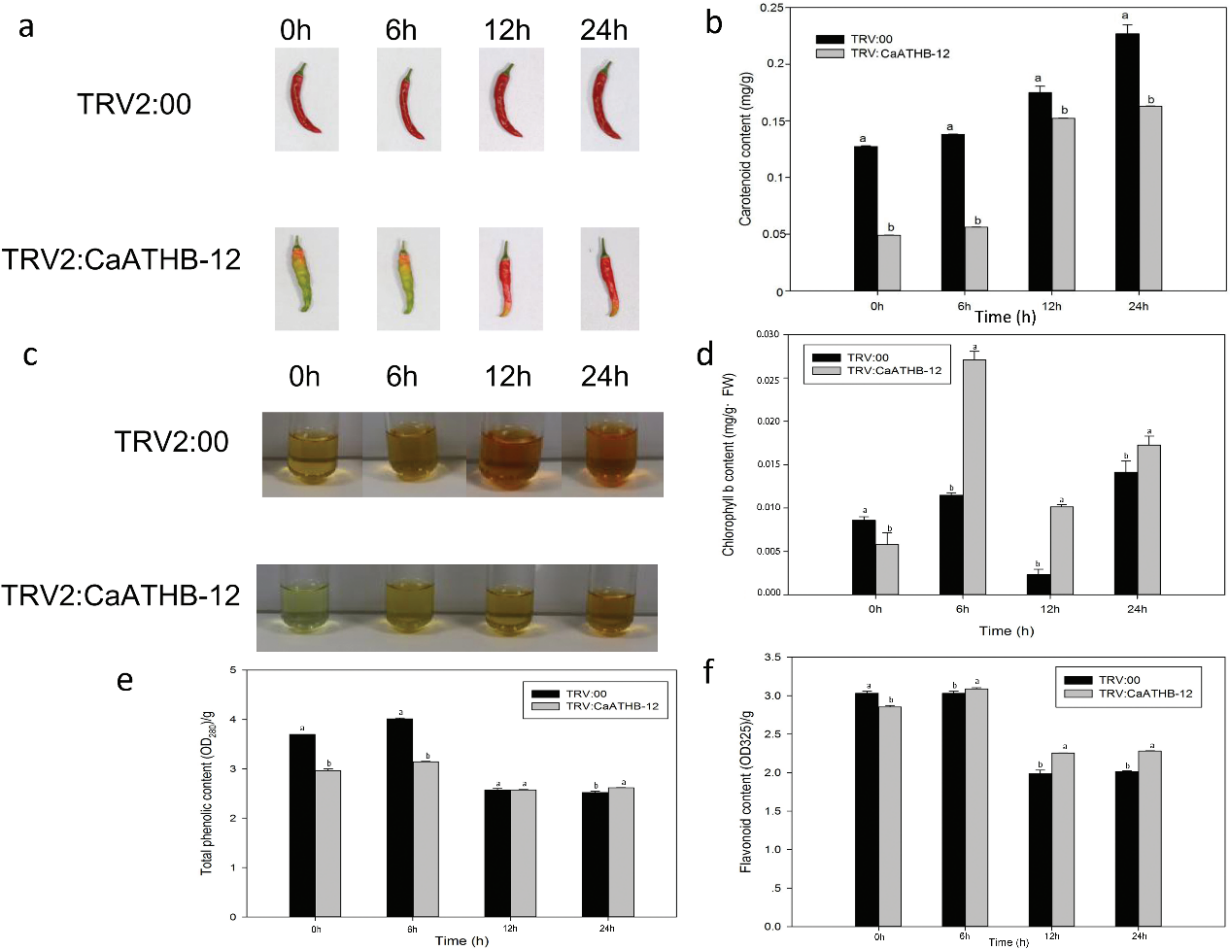
Effect of cold stress on carotenoid content in the fruits of pepper. The samples at the detached fruits were collected at different time points (0, 6, 12, and 24 h), and detached fruits were exposed to 4°C. (a) Phenotypic change after cold treatment is between TRV2:00 and TRV2:*CaATHB-12*. (b) Carotenoid content changes after cold treatment. (c) Pigment content changes. (d) Chlorophyll b content changes after cold treatment. (e) Total phenolic content changes. (f) Flavonoid content changes after cold treatment. Mean values and SDs for three replicates are shown. Error bars represent SD for three biological replicates, and the letters show the significance level at *α* = 0.05.

**Fig. 4:**
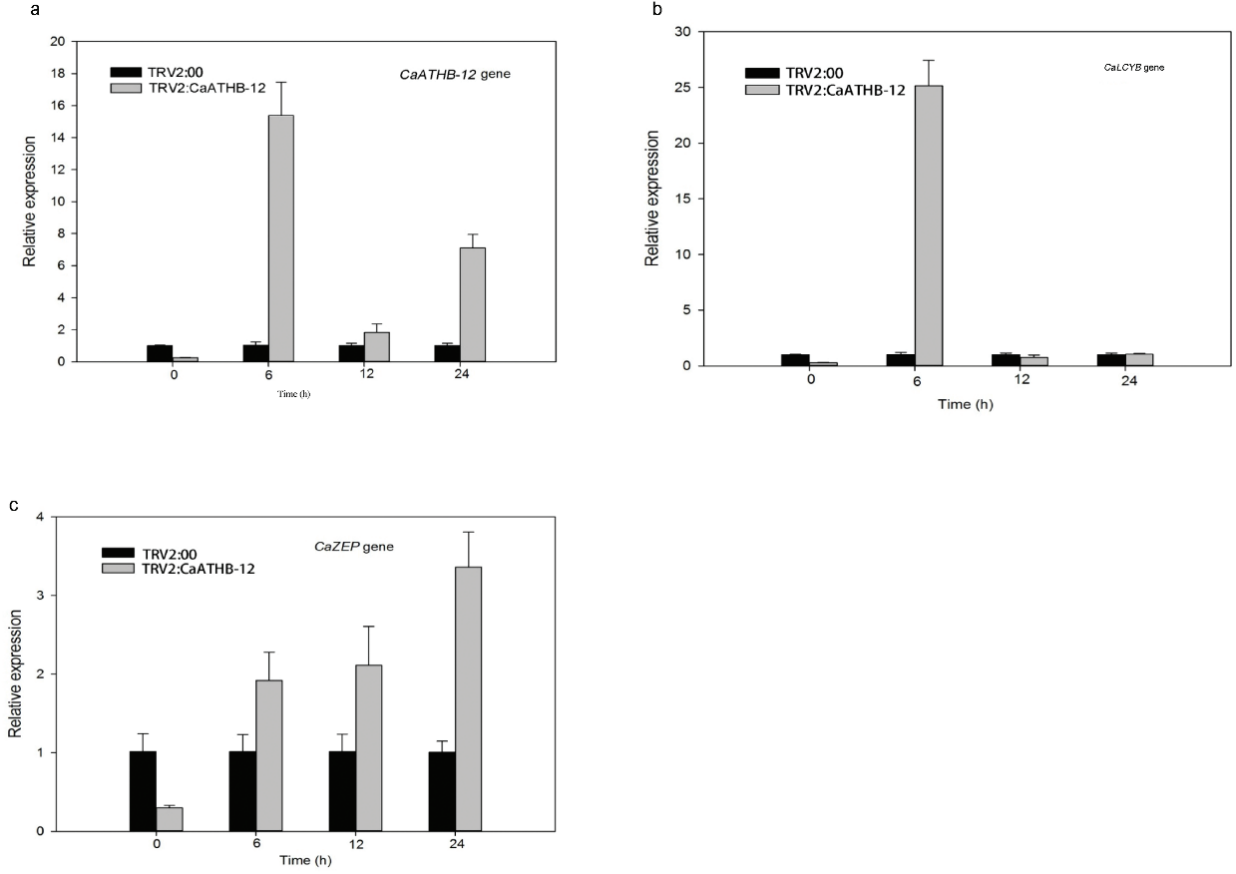
Expression profiles of regulatory genes related to carotenoid synthesis in response to cold stress. (a–c) Detached fruits were exposed to 4°C, and levels in the expression of (a) *CaATHB-12*, (b) *CaLCYB*, and (c) *CaZEP* were investigated by qRT-PCR.

**Fig. 5:**
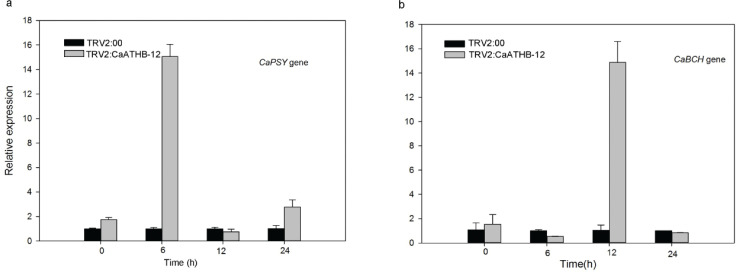
Expression profiles of regulatory genes related to carotenoid synthesis in response to cold stress. (a–b) Detached fruits were exposed to 4°C, and levels in the expression of (a) *CaPSY* and (b) *CaBCH* were investigated by qRT-PCR.

To investigate the effect of low temperature on *CaATHB-12* in isolated pepper fruits, we first determined the changes in carotenoid content under low temperature stress (Fig. 3). Figure 3a shows the performance of pepper after cold stress, and the color was deepened. Correspondingly, the carotenoid content was also changed (Fig. 3b). The carotenoid content increased significantly, but the levels remained significantly lower than the control of TRV2:00 (Fig. 3c). Further, the expressions of carotenoid biosynthesis-related genes were measured by qRT-PCR (Figs. 3 and 4). Chlorophyll b resisted the damage of fruits at low temperature, and the chlorophyll b contents of the 6 h-silenced fruits were significantly higher than the control fruits (Fig. 3d). At 12 h, the total content of total phenols and flavonoids was significantly higher than the control (Fig. 3e, f), which indicated that the silenced pepper fruits had a more total phenols and flavonoids to resist the effect of low temperature on the fruits. In the pepper of TRV2:*CaATHB-12*, the *CaATHB-12* expression was significantly upregulated at 6 h and then decreased at 12 and 24 h, but the expression levels of all the genes were higher than that at 0 h (Fig. 4a). Similarly, the expression of *CaLCYB* gene was also significantly increased first at 6 h and then decreased, which followed a similar trend as of *CaATHB-12* expression (Fig. 4b). In addition, we also found that the expression of *CaZEP* gene in the silenced fruit at 0 h was lower than that of the control, and then began to rise in the following time points, and the expression level showed an upward trend; both were higher than the control at 6, 12, and 24 h (Fig. 4c). The expression of *CaPSY* and *CaBCH* genes was different from those of *CaZEP*. At 0 h, the transcriptional levels of the two genes in the silenced fruit were higher than the control fruits, where the expression of *CaPSY* gene reached to a maximum at 6 h, while the *CaBCH* gene reached to a maximum at 12 h, and then the expression decreased (Fig. 5). These data indicated that the *CaATHB-12* gene and the carotenoid synthesis-related genes corresponded to the low temperatures, and the carotenoid content increased significantly with the prolongation of the low-temperature treatment.

### Effect of cold stress on antioxidants

**Fig. 6:**
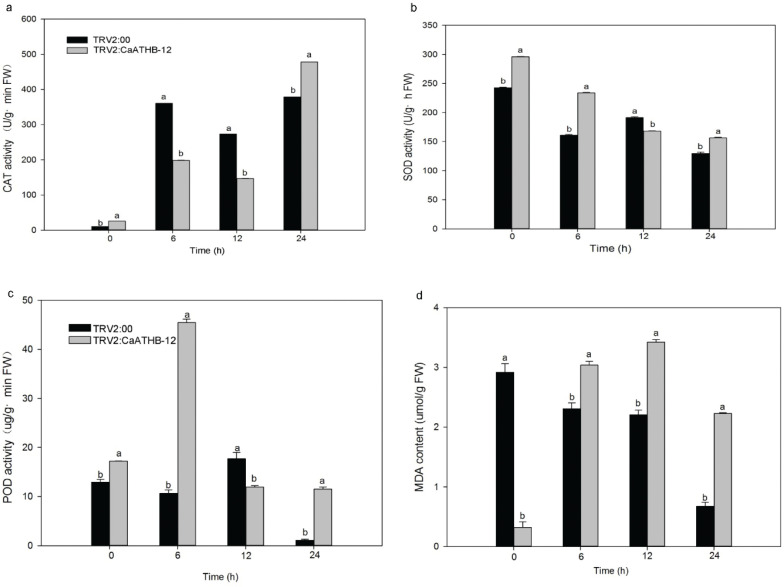
The effect of *CaATHB-12* silencing on antioxidant enzymes under cold stress in pepper. (a) CAT activity; (b) SOD activity; (c) POD activity; (d) MDA content. The fruit was sampled at about 1 month after infected. Triplicates were conducted for this experiment, the error bar represented SD of three triplicates, and the letters show the significance level at *α* = 0.05.

After low-temperature treatment, the CAT content in the peppers of TRV2:*CaATHB-12* showed a significant increase and reached the maximum at 24 h. Compared with the control, the CAT activity at 24 h was significantly higher than the control (Fig. 6a). The SOD activity showed a downward trend, but the activity of SOD in the pepper of TRV2:*CaATHB-12* was significantly higher than that of the control at 0, 6, and 24 h (Fig. 6b). The activity of POD in the pepper of TRV2:*CaATHB-12* increased first at 6 h and then decreased in the following time point at 12 h and 24 h. Interestingly, the activity of POD enzyme returned to the lower level after 24 h as compared with 0 h, whereas the activity of POD enzyme in the fruits of silenced pepper was significantly higher at the corresponding time point (0, 6, and 24 h), except for 12 h (Fig. 6c). MDA is one of the important indicators for measuring the membrane lipid peroxidation. At 0 h, MDA content was significantly lower than the control, whereas, with the prolongation of the low-temperature treatment time, the MDA content of the TRV2:*CaATHB-12* increased rapidly as compared with the control. There was a significant increase in the MDA content at 6, 12, and 24 h. Although the MDA content decreased at 24 h, there was a significant upregulation relative to the MDA content at 0 h (Fig. 6d).

### The effect of CaATHB-12-silenced gene under cold stress on the expression of the antioxidant genes in the detached fruits of pepper

**Fig. 7:**
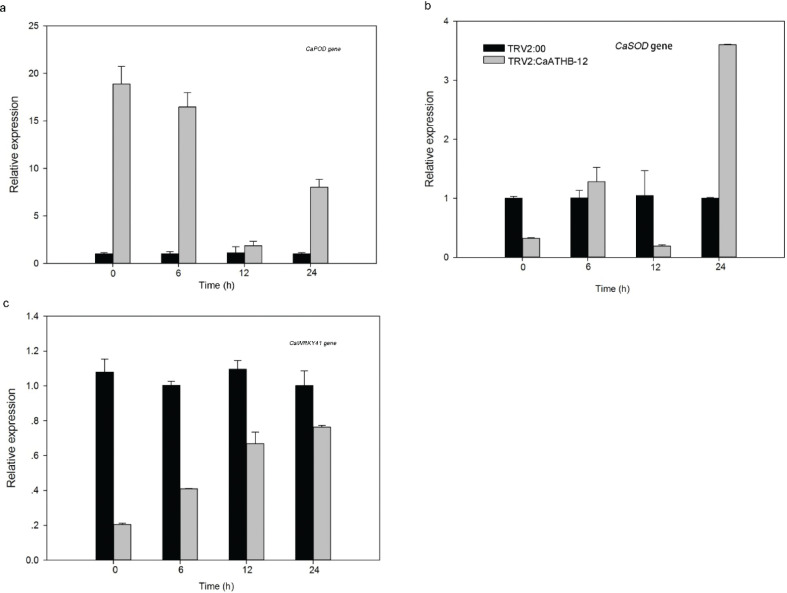
The effect of *CaATHB-12* silencing on antioxidant gene expression and low-temperature response gene expression under cold stress in pepper. (a) *CaPOD* expression; (b) *CaSOD* expression; (c) *CaWRKY41* expression. The fruit was sampled at about 1 month after infected. Triplicates were conducted for this experiment, the error bar represented SD of three triplicates, and the letters show the significance level at *α* = 0.05.

*CaPOD*, one of the antioxidant enzyme genes, was consistently upregulated during the treatment as compared with the respective control TRV2:00 fruits (Fig. 7a). The relative expression of *CaSOD* varied where the relative expression reached a peak at 24 h. Under cold stress treatment, the overall trend showed an upward trend (Fig. 7b). The expression of *CaWRKY41* gene, an important low-temperature response gene, increased with the low-temperature treatment, but it was significantly lower than the control group (Fig. 7c).

### Effect of CaATHB-12 overexpression in the transgenic Arabidopsis

**Fig. 8:**
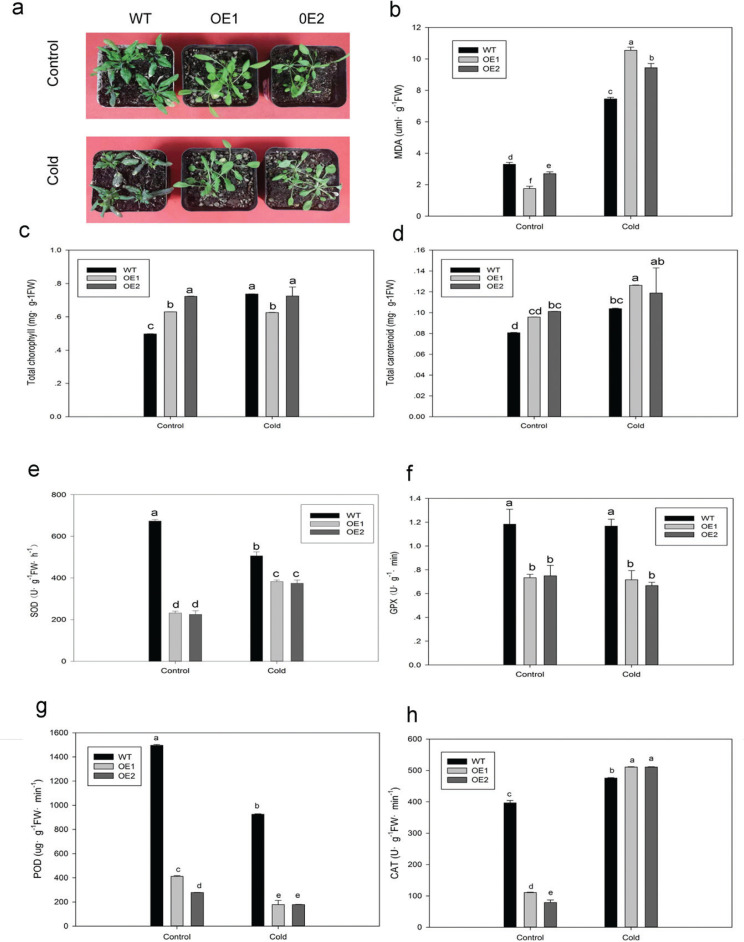
Overexpression of the *CaATHB-12* gene reduces tolerance to cold stress. (a–h) Phenotype, MDA content, total chlorophyll content, total carotenoid content, SOD, GPX, POD, and CAT activity of WT and *CaATHB-12*-OE *Arabidopsis* lines (OE1 and OE2) at 4°C for 24 h. Seedlings grown at 22°C were used as the control. Data are means with standard deviations of three biological replicates. Different letters denote statistical significance at *P* ≤ 0.05.

*Arabidopsis* transgenic lines overexpressing *CaATHB-12*, OE1 (over expression), and OE2 were used to perform abiotic stress treatments. Although no visible difference was observed between *CaATHB-12*-OE lines and wild-type (WT) *Arabidopsis* plants under normal growth conditions, a significant increase in the levels of *CaHSP22.0* expression was observed in the transgenic plants (OE1 and OE2) (Supplementary Fig. 3). After cold treatment at 4°C for 24 h, severe wilting symptoms were observed in the *CaATHB-12*-OE seedlings. Interestingly, we did not observe any changes in morphology in the WT plants (Fig. 8a). The total carotenoid content of the *CaATHB-12*-OE seedlings was significantly higher than WT (Fig. 8d). However, no significant change was observed in the total chlorophyll content in *CaATHB-12*-OE plants (Fig. 8c). The MDA content in OE lines was higher than that of the WTs (Fig. 8b). Similar results to MDA content were observed in CAT activity as well, but both have high activities compared within WT (Fig. 8h). Interestingly, after treated with cold stress, activities of these antioxidant enzymes SOD, GPX, and POD in the *CaATHB-12*-OE lines showed significant decrease as compared with the WT plants (Fig. 8e–g).

**Fig. 9:**
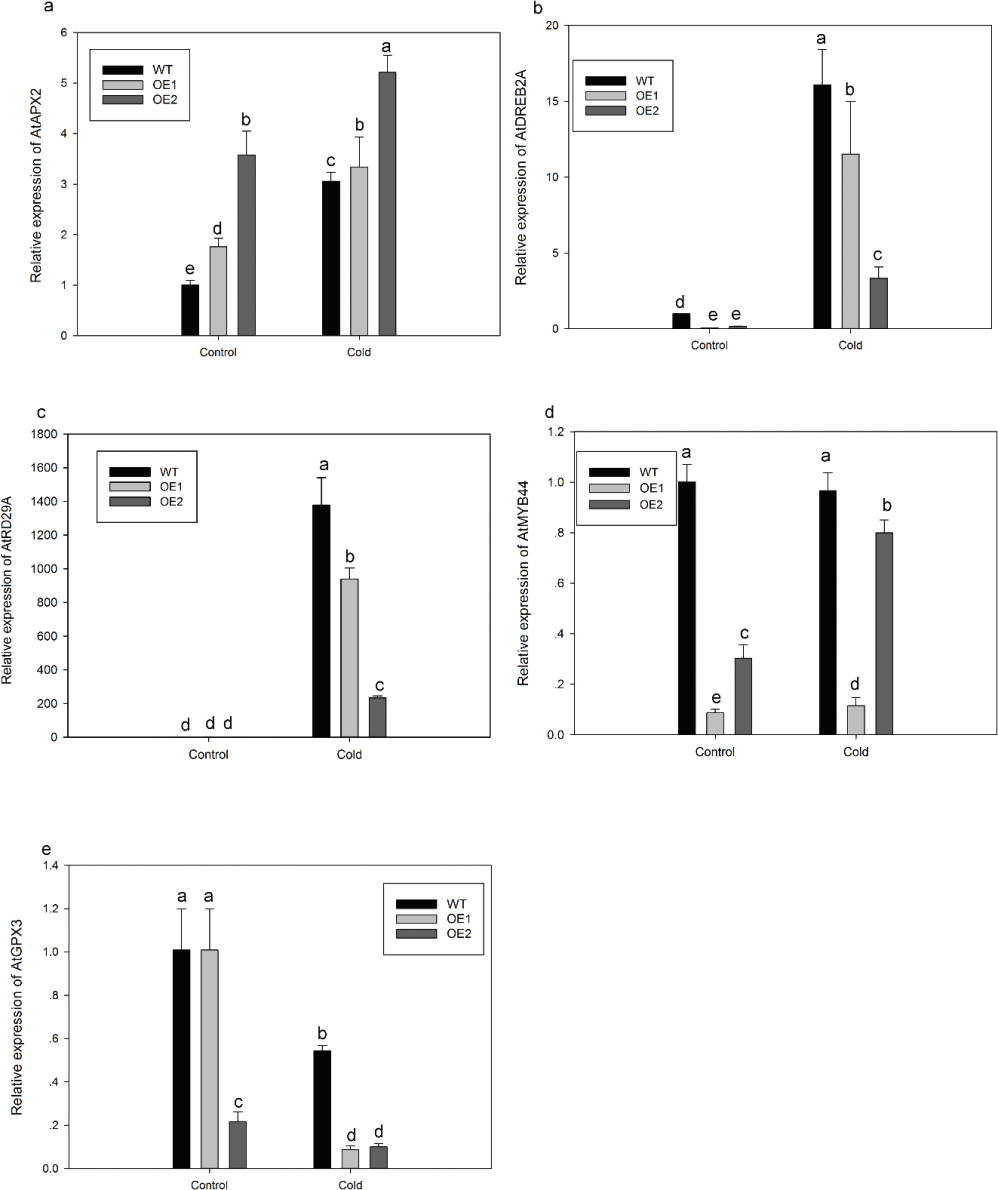
Relative expression levels of related genes in WT and *CaATHB-12*-OE lines under cold stress. Relative expressions of (a) *ATAPX2*, (b) *ATDREB2A*, (c) *ATRD29A*, (d) *ATMYB44*, and (e) *ATGPX3*. Bars show the standard deviation of expression levels from three biological replications. Different letters denote statistical significance at *P* ≤ 0.05.

Next, we further examined the expression patterns of stress-responsive genes (*AtRD29A*, *AtMYB44*, *AtDREB2A*, *AtAPX2*, and *ATGPX3*) after treatment with 4°C for 24 h. The results showed that under normal conditions, these stress-responsive genes displayed low transcript levels in OE-lines and WT (Fig. 9b, c). However, under the cold stress conditions, the expression levels of all the mentioned genes were upregulated in *CaATHB-12*-OE lines, but both the enhanced folds and transcript abundance were also lower in the transgenic lines than in WT plants (Fig. 9b, c). Similarly, after cold stress treatment, the relative expression levels of *AtDREB2A*, *AtMYB44*, *AtRD29A*, and *ATGPX3* were significantly lowered in *CaATHB-12*-OE plants as compared with the WT, except the *AtAPX2* gene (Fig. 9).

## Discussion

It is widely reported that stress conditions result in an increase in the ROS levels and also stimulate the action of biological antioxidants (49, 50). Carotenoids, as an important antioxidant, are supported by their ubiquity in the nature. They are the main dietary source of vitamin A in humans (51), which has a link between plants and their environment and plays an important role in the improvement of tolerance to stress by inhibition of ROS (52, 53). Studies on plant antioxidant responses indicate that a crucial part of the antioxidant network operates in cells and their action shows a high level of interdependence that can be influenced by plant cold tolerance (50). Carotenoids may also act as powerful antioxidants, protecting fruit tissues from different stresses (54). Moreover, lycopene-induced chilling tolerance in grapefruits appears to be directly related to an enhancement of the singlet oxygen scavenging capacity (55). Generally, cells lacking carotenoids are much easier to be attracted by ROS (56). The transcriptional levels of carotenoid biosynthesis genes usually show upregulation when plants are exposed to adversities, thereby leading to higher levels of carotenoids (57). In the current study, we characterized contribution of a novel transcription factor gene, *CaATHB-12*, in response to cold stress. As the subcellular localization of proteins is closely related to their function, most of the previously obtained HD-Zip family members are located in the nucleus (58), for example, *Athb-12* in *Arabidopsis* in the nucleus (59), and then *Oshox12*, *Oshox14*, *Oshox22*, and *ZmHDZ1* are nuclear-localized proteins (60–63). Consistent with the known function of TFs, the GFP-tagged fusion constructs indicated that *CaATHB-12* has nuclear-localized proteins (Fig. 1). Silencing of the *CaATHB-12* gene leads to the decreased carotenoid contents. The sunflower HD-Zip transcription factor *HAHB4* resulted in the contents of chlorophylls a and b, and carotenoids were decreased in *Arabidopsis* transgenic plants, with downregulated the chlorophyll-binding proteins (27). In navel orange, low temperature (°C) significantly enhanced the expression levels of *CaPSY*, *CaLCYB*, and *CaZEP* genes compared with 20°C (64). The *CaPSY* gene acts as one of the most important rate-limiting enzyme in the carotenoid metabolic pathway, and the *CaPSY* gene responds to posttranscriptional feedback regulation of environmental stresses such as hypothermia, high lights, salt, and ABA (65). Overexpression of the *CaPSY* genes in the *Arabidopsis*-enhanced tolerance to salt stress (66). As a non-enzymatic antioxidant, carotenoids play an important role in scavenging the ROS. Many members of the HD-ZIP I subfamily respond to environmental stresses and also regulate the plant growth and development (9). *ATHB-12* is reported in the *Arabidopsis* in response to ABA, and to drought (67) and salt stress (68). We reported that TRV2:*CaATHB-12* silencing in pepper has less carotenoid accumulation as compared with the control pepper plants. The silencing of *MdHB1* in apple ‘Granny Smith’ fruit activated the expression of *MdDFR* and *MdUFGT* and also the anthocyanin biosynthesis, whereas its overexpression reduced the flesh content of anthocyanin in ‘Ballerina’ (red-fleshed apple) (25). On the other hand, the carotenoid contents in the overexpression of *CaATHB-12* lines were higher than the WT *Arabidopsis* plants (Figs. 3 and 8d); however, after low-temperature treatment, the carotenoid content significantly increased (Fig. 3b). Thereafter, we also measured the carotenoid biosynthesis-related genes expression. Interestingly, these genes, such as *CaPSY* and *CaLCYB*, first significantly upregulated at 6 h and then returned to the initial state (Figs. 4b and 5a). Some reports indicated that the overexpression of the *CaPSY* genes leads to the accumulation of carotenoids in *Arabidopsis* (69).

The miracle of carotenoid biosynthesis in the organism is closely related to the regulation of the metabolic pathways. The regulation of the *CaLCYB* gene produces β-carotene, which has an effect on the production of carotenoids. The low-temperature condition of β-carotene is increased in *Arabidopsis* (65). Fungus *Neurospora crassa* has a higher transcripts abundance of carotenoid synthesis-related genes at low temperatures (70), and chilling damage reduces lycopene contents in tomato and lycopene act as a carotenoid (71). *CaZEP* is involved in carotenoid biosynthesis and lutein cycling, which removed excess ROS, is a mechanism of plant protection (72), acts as a strong antioxidant to the extreme environments, and works on light protection. The *ZEP* genes play an important role in protecting plants photosynthetic systems from photochemical damage under extreme environmental conditions (73). While participating in drought and extreme stress processes (74), higher expression levels of the *ZEP* genes are closely related to the regulation of the xanthophyll cycle (75). Our report indicated that the *CaZEP* gene was upregulated during the 24 h cold stress period (Fig. 4c), suggesting that the *CaZEP* gene played a role in response to the low temperature. The similar report was found in *alfalfa* where the upregulated expression of *MsCaZEP* enhanced tolerance to environmental stresses (76).

During the process of evolution, plants have evolved a series of complex responsive mechanisms to adapt to various environmental stresses. Antioxidant enzymes promote oxidative stress in the cells and protect against cellular damage caused by the ROS (77, 78). Plants have complex antioxidant systems for the scavenging of ROS, and several important antioxidant enzymes play an important role (79). Higher antioxidant enzyme activities have a stronger ability to scavenge ROS, which has a better protective effect on the plants (4). After low-temperature treatment, POD was significantly upregulated at 6 h (Fig. 6c) in the silenced pepper fruits, whereas there was no significant change in the control, and low temperature also increased the POD activity in cucumber leaves (80). At the same time, the overall activity of CAT showed an upward trend (Fig. 5a). It was reported in pepper that the CAT activity decreased within 24 h after low-temperature treatment, suggesting that the protective mechanism of active oxygen scavenging in the short term mainly relied on POD and SOD enzyme activities (81). Another report pointed out that increased CAT activity under low-temperature induction may suggest that H_2_O_2_ may be removed, thus avoiding the formation of hydroxyl radicals and cold-induced damage (82, 83). The SOD content decreased overall, but the SOD activity was significantly higher at 0, 6, and 24 h than the control peppers, (Fig. 6b). In pepper, exposure to 8°C for 24 h, oxidative stress was induced (84); the same report was found in cucumber and wheat seedlings, where low temperature significantly induced the SOD enzyme activity (4, 85), which was also reported in rice (86). The MDA content is used as one of the important indicators for monitoring the membrane lipid peroxidation under stress conditions (87); low-temperature increased the MDA contents (83). Our report indicated that the MDA content in the control group was higher than that of the silenced fruit at 0 h, while the activities of SOD, POD, and CAT were higher than that of the control (Fig. 6d), indicating that more antioxidant enzymes in the silenced fruit cleared the active oxygen. The MDA content was low, and the silencing of *CaATHB-12* resulted in better tolerance to low temperature than the control pepper plants.

Next, we further examined the expression patterns of stress-responsive genes (*CaSOD*, *CaPOD*, and *CaWRKY41*) after treatment with cold stress for 24 h. The *CaSOD* and *CaPOD* genes strongly responded to low temperature and oxidative stress responses (80, 83). Our results also showed that the highest expression of 0 h *CaPOD* was compared with other time points. Under low-temperature treatments, the overall increase of *CaSOD* gene expression peaked at 24 h (Fig. 7b). Reports indicated that the overexpression of *SOD* gene enhanced tolerance to low temperatures in *rapeseed* (88). However, the expression level of *CaPOD* gene was significantly higher than that of the control at each time point (Fig. 7a). The upregulated expression of *CaPOD* gene promoted POD enzyme activity, suggesting the role in low-temperature response. As a low-temperature response gene, the *CaWRKY41* gene responded to a low temperature and was upregulated with the prolongation of cold stress (Fig. 7c). In *tobacco*, the expression of *WRKY41* was upregulated at low temperatures (89). Further, our study suggested that silencing of *CaATHB-12* gene has a positive association with the upregulation of antioxidant enzyme genes, including *CaPOD* and *CaSOD* genes, which resulted in enhanced tolerance of pepper at low temperatures.

To further understand the biological functions of *CaATHB-12* in plant response to cold stress, we employed transgenic approach in the *Arabidopsis* and found that the *CaATHB-12* overexpression in *Arabidopsis* seedlings exhibited more wilted leaves than WT seedlings. It was also reported earlier in *Arabidopsis* that oxidative stress was induced at 4°C (90). A previous study divulged that overexpression of the *ATHB12* induced the formation of larger leaves with enlarged cells of higher-ploidy levels, suggested that *ATHB12* positively regulates the cell expansion in *Arabidopsis* (91). Similarly, in other crop species, the overexpression of *OsHox22*, a gene belongs to rice HD-Zip I family, decreased the transgenic rice tolerance to environmental stresses, which indicated that the *OsHox22* played the roles as negative regulators in rice (62). Overexpression of *ZmHDZ1* in rice as well showed a similar trend (63). Generally, *AtDREB2A* can be induced by low temperature (92), and the expression of *RD29A* can be used as a criterion for plant development and stress resistance (93, 94). *AtAPX2*, *AtGPX3*, and *AtMYB44* responded to adverse stress situations (95). In our results, the expression of *AtRD29A*, *AtRDEB2A*, *AtGPX3*, and *AtMYB44* transcripts abundance and activities of antioxidant enzymes in *CaATHB-12*-OE lines were lower than in WT plants under cold stresses conditions (Figs. 8 and 9). This suggests that these genes certainly decreased the stress tolerance of *CaATHB-12*-OE lines by not checking the ROS level. Normally, there is a proper balance between generation and scavenging of ROS in control and stress tolerance and is regulated by complex signal transduction pathways (96, 97). This situation could be due to the transgenic lines have less antioxidant enzyme activities and higher MDA content, leading to the aggravation of membrane lipid peroxidation (98). Taken together, our results suggested that *CaATHB-12* may be involved in plant cold stress tolerance by modulating the expression level of stress-related genes.

## Conclusion

In this study, we found that the *CaATHB-12* gene is involved in the regulation of the fruit color in the pepper AA3, and the carotenoid content of the silenced pepper fruits was significantly lower than the control fruit. Further, under the cold stress, the increased contents of carotenoids, flavonoids, and phenolic compounds were due to the upregulation of the carotenoid biosynthesis-related genes. Moreover, the *CaATHB-12*-silenced fruit led to a higher level of antioxidant enzyme activities and transcript abundance of the antioxidant enzyme-related genes. While the overexpression of *CaATHB-12* increased the content of carotenoid in the normal condition, the ability of ROS scavenging was impaired under cold stress. This study provides a better understanding of the role of *CaATHB-12* in resisting low-temperature stress during the development of pepper fruit color. *CaATHB-12* was also involved in cold stress tolerance through scavenging of the ROS. This study provides a basis for further research on the role of this vital gene in the carotenoid biosynthesis and cold stress response in other important crops species.

## Supplementary Material

A novel gene, *CaATHB-12*, negatively regulates fruit carotenoid content under cold stress in *Capsicum annuum*
