# Supplementary material for: A novel gene, CaATHB-12, negatively regulates fruit carotenoid content under cold stress in Capsicum annuum
Source: Food Nutr Res. 2020 Dec 28;64:10.29219/fnr.v64.3729. doi: 10.29219/fnr.v64.3729 (PMC7778427; doi:10.29219/fnr.v64.3729)
Supplement: A novel gene, CaATHB-12, negatively regulates fruit carotenoid content under cold stress in Capsicum annuum [file FNR-64-3729-s001.docx]

| **id** | **site** | **distance** | **identity** | **comments** |
| --- | --- | --- | --- | --- |
| HAT5_ARATH | nucl | 197.438 | [27.2059%](https://www.genscript.com/tools/wolf-psort/detail?file=15851393702021.alignment1.html#HAT5_ARATH) | [[Uniprot]](https://www.genscript.com/tools/wolf-psort/detail?file=http://www.uniprot.org/entry/Q02283) SWISS-PROT45:Nuclear. |
| At3g26744.1 | nucl | 223.08 | [12.9555%](https://www.genscript.com/tools/wolf-psort/detail?file=15851393702021.alignment1.html#At3g26744.1) | [[Arath]](https://www.genscript.com/tools/wolf-psort/detail?file=http://arabidopsis.org/servlets/mapper?value=At3g26744.1&action=search) |
| HFR1_ARATH | nucl | 245.111 | [12.6712%](https://www.genscript.com/tools/wolf-psort/detail?file=15851393702021.alignment1.html#HFR1_ARATH) | [[Uniprot]](https://www.genscript.com/tools/wolf-psort/detail?file=http://www.uniprot.org/entry/Q9FE22) SWISS-PROT45:Nuclear. |
| AG_TOBAC | nucl | 247.752 | [10%](https://www.genscript.com/tools/wolf-psort/detail?file=15851393702021.alignment1.html#AG_TOBAC) | [[Uniprot]](https://www.genscript.com/tools/wolf-psort/detail?file=http://www.uniprot.org/entry/Q43585) SWISS-PROT45:Nuclear. |
| HY5_ARATH | nucl | 250.134 | [14.876%](https://www.genscript.com/tools/wolf-psort/detail?file=15851393702021.alignment1.html#HY5_ARATH) | [[Arath]](https://www.genscript.com/tools/wolf-psort/detail?file=http://arabidopsis.org/servlets/mapper?value=At5g11260.1&action=search) [[Uniprot]](https://www.genscript.com/tools/wolf-psort/detail?file=http://www.uniprot.org/entry/O24646) SWISS-PROT45:Nuclear. Evidence:IDA Pubmed:[9367981](https://www.genscript.com/tools/wolf-psort/detail?file=http://www.ncbi.nlm.nih.gov/entrez/query.fcgi?cmd=Retrieve&db=pubmed&dopt=Abstract&list_uids=9367981),[9596629](https://www.genscript.com/tools/wolf-psort/detail?file=http://www.ncbi.nlm.nih.gov/entrez/query.fcgi?cmd=Retrieve&db=pubmed&dopt=Abstract&list_uids=9596629) |
| PIF4_ARATH | nucl | 251.383 | [11.3953%](https://www.genscript.com/tools/wolf-psort/detail?file=15851393702021.alignment1.html#PIF4_ARATH) | [[Arath]](https://www.genscript.com/tools/wolf-psort/detail?file=http://arabidopsis.org/servlets/mapper?value=At2g43010.1&action=search) [[Uniprot]](https://www.genscript.com/tools/wolf-psort/detail?file=http://www.uniprot.org/entry/Q8W2F3) SWISS-PROT45:Nuclear. Evidence:IDA Pubmed:[12006496](https://www.genscript.com/tools/wolf-psort/detail?file=http://www.ncbi.nlm.nih.gov/entrez/query.fcgi?cmd=Retrieve&db=pubmed&dopt=Abstract&list_uids=12006496) |
| PRH_PETCR | nucl | 254.154 | [7.35294%](https://www.genscript.com/tools/wolf-psort/detail?file=15851393702021.alignment1.html#PRH_PETCR) | [[Uniprot]](https://www.genscript.com/tools/wolf-psort/detail?file=http://www.uniprot.org/entry/P48786) SWISS-PROT45:Nuclear. |
| SR14_ARATH | cyto | 254.632 | [13.2231%](https://www.genscript.com/tools/wolf-psort/detail?file=15851393702021.alignment1.html#SR14_ARATH) | [[Uniprot]](https://www.genscript.com/tools/wolf-psort/detail?file=http://www.uniprot.org/entry/O04421) SWISS-PROT45:Cytoplasmic. |
| At3g47690.1 | cysk | 255.141 | [12.3188%](https://www.genscript.com/tools/wolf-psort/detail?file=15851393702021.alignment1.html#At3g47690.1) | [[Arath]](https://www.genscript.com/tools/wolf-psort/detail?file=http://arabidopsis.org/servlets/mapper?value=At3g47690.1&action=search) sequence changed in 2004 |
| TGAA_TOBAC | nucl | 260.622 | [15.5989%](https://www.genscript.com/tools/wolf-psort/detail?file=15851393702021.alignment1.html#TGAA_TOBAC) | [[Uniprot]](https://www.genscript.com/tools/wolf-psort/detail?file=http://www.uniprot.org/entry/P14232) SWISS-PROT45:Nuclear. |
| AG_ARATH | nucl | 263.055 | [10.2767%](https://www.genscript.com/tools/wolf-psort/detail?file=15851393702021.alignment1.html#AG_ARATH) | [[Uniprot]](https://www.genscript.com/tools/wolf-psort/detail?file=http://www.uniprot.org/entry/P17839) SWISS-PROT45:Nuclear. |
| AGL5_ARATH | nucl | 271.408 | [12.4%](https://www.genscript.com/tools/wolf-psort/detail?file=15851393702021.alignment1.html#AGL5_ARATH) | [[Uniprot]](https://www.genscript.com/tools/wolf-psort/detail?file=http://www.uniprot.org/entry/P29385) SWISS-PROT45:Nuclear. |
| AGL1_ARATH | nucl | 272.891 | [10.4839%](https://www.genscript.com/tools/wolf-psort/detail?file=15851393702021.alignment1.html#AGL1_ARATH) | [[Uniprot]](https://www.genscript.com/tools/wolf-psort/detail?file=http://www.uniprot.org/entry/P29381) SWISS-PROT45:Nuclear. |
| AG_BRANA | nucl | 275.874 | [10.2767%](https://www.genscript.com/tools/wolf-psort/detail?file=15851393702021.alignment1.html#AG_BRANA) | [[Uniprot]](https://www.genscript.com/tools/wolf-psort/detail?file=http://www.uniprot.org/entry/Q01540) SWISS-PROT45:Nuclear. |

**Table S3. Predicted subcellular localization of *CaATHB-12* based on online tools.**
